# Changes of local microenvironment and systemic immunity after acupuncture stimulation during inflammation: A literature review of animal studies

**DOI:** 10.3389/fneur.2022.1086195

**Published:** 2023-01-11

**Authors:** Wei-Lien Yu, Ji-Yeun Park, Hi-Joon Park, Seung-Nam Kim

**Affiliations:** ^1^College of Korean Medicine, Dongguk University, Goyang-si, Republic of Korea; ^2^College of Korean Medicine, Daejeon University, Daejeon, Republic of Korea; ^3^College of Korean Medicine, Kyung Hee University, Seoul, Republic of Korea

**Keywords:** acupuncture, inflammation, microenvironment, literature review, mast cell, nervous system

## Abstract

An increasing number of studies have demonstrated the underlying mechanisms by which acupuncture therapy mediates both local and systemic immunomodulation. However, the connection between alterations in the local microenvironment and the resulting change in systemic immunity remains unclear. In this review, we focus on cell-specific changes in local immune responses following acupuncture stimulation and their link to systemic immune modulation. We have gathered the most recent evidence for chemo- and mechano-reactive changes in endothelial cells, neutrophils, macrophages, and mast cells in response to acupuncture. Local signaling is then related to the activation of systemic neuro-immunity including the cholinergic, adrenal, and splenic nervous systems and pain-related neuromodulation. This review aims to serve as a reference for further research in this field.

## 1. Introduction

Acupuncture involves inserting thin needles into the skin or tissues to cure various physiological dysfunctions. Acupoints are specific superficial anatomic points that are thought to be linked with the internal organs. These points are defined using certain anatomical landmarks, each of which is prescribed for different pathological conditions. For example, the acupoint ST36 is located on the tibialis anterior muscle and 3/16 down the relative length of the tibia (1). Clinical studies have supported the application of ST36 stimulation in stroke rehabilitation, diabetes management, and post-operative ileus prevention (2–4). Different acupoints are distributed in various cell types and populations, as well as in different nerve circuits. This likely explains acupoint-specific effects in clinical practice. Despite its long history of use, the biological mechanisms of acupuncture therapy are only beginning to emerge. The practice is gaining respect as a valid therapeutic option for many conditions. Clinically, manual acupuncture (MA) and electroacupuncture (EA) have been used as main acupuncture methods. EA is a recently developed method, which combined MA with electrical stimulation; it allows for easier manipulation of stimulus strength.

Various mechanisms have been proposed to explain acupuncture-mediated immunomodulation. Acupuncture has been shown to downregulate the toll-like receptor (TLR)-initiated inflammatory signaling pathway in various inflammatory disease models (5–7). The upregulation of anti-inflammatory cytokines, particularly interleukin (IL)-10, promoted M1–M2 macrophage transformation, which is crucial for inflammation resolution (8). In chronic inflammatory disease models, acupuncture balanced the helper T cell (Th)1/Th2 population to treat immune malfunction (9). The nervous system also participates in local inflammation *via* paracrine or endocrine signaling. Different signaling molecules, such as neurotransmitters and cytokines, have been shown to contribute to crosstalk between neurons and immune cells (10). Acupuncture has also been associated with alterations in the hypothalamic–pituitary–adrenal (HPA) axis, a neural pathway mediating systemic immunity (11).

Acupuncture-mediated immunomodulatory mechanisms are classified largely into two categories: the immediate local response initiated by the innate immune system and the systemic immunity regulated by neural pathways. First, we examined the local cell signaling pathways associated with acupuncture-mediated immunomodulation. These signaling pathways have been divided into the ligand—and mechanically stimulated—elements of the sterile inflammatory response. The second aspect of this review summarizes the current knowledge bridging the fields of immunology and neurology that overlap with the described cellular mechanisms to explain the observed long-term benefits of acupuncture. This review will provide a theoretical basis for the unionized understanding of future studies in these fields.

## 2. Main text

### 2.1. Local induction of inflammation

#### 2.1.1. The local inflammatory response—Ligand-mediated

The immediate host response to needle insertion can be explained with the term “the sterile inflammatory response”—inflammation in the absence of infection (12). Damage-associated molecular patterns (DAMPs) are released after tissue injury. Subsequently, immune cells expressing pattern recognition receptors (PRRs) are activated. Activated immune cells, including mast cells and macrophages, release cytokines and chemokines that attract more leukocytes.

##### 2.1.1.1. Endothelial cells

Endothelial cells (ECs) are gatekeepers that control the exchange of fluids and materials between blood and the surrounding tissues (13). Under physiological conditions, ECs maintain blood fluidity, blood flow, and vascular permeability through intrinsic signals from mechanoreceptors and extrinsic signals from the autonomous nervous system. Vascular permeability and tone are determinants of substance exchange across blood vessels. Nitric oxide (NO) is a major vasodilator formed mainly by endothelial NO synthase. Certain acupoints are more effective in increasing NO levels (14). The release of NO through EC signaling increases the exchange rate across vessel walls and provides a more oxygen- and energy-rich microenvironment for tissue repair. Metabolic reprogramming occurs during inflammation, which influences immunity. Recent studies have suggested that the metabolic shift from glycolysis to oxidative phosphorylation is a key factor in the successful activation of M2 macrophages (15). Classical symptoms of inflammation include pain, heat, redness, and swelling. Acupuncture induces immune cell infiltration; while pain and swelling are less commonly observed following acupuncture therapy. Further immune profiling and immunophenotyping may decipher the acupoint-specific regulation of inflammation in different pathogeneses.

##### 2.1.1.2. Neutrophils

The first leukocytes to be recruited to an inflammatory site are neutrophils. In sterile injury, their major role is tissue repair (12, 15). Neutrophils remove necrotic debris, release growth factors, and deplete chemoattractants to balance the inflammatory reaction. The anti-inflammatory effects of acupuncture have been extensively characterized in animal models. These studies found that the most commonly upregulated cytokines in sterile injury include tumor growth factor-beta (TGF-β) and IL-10, while toxic effectors such as reactive oxygen species (ROS) and proteolytic enzymes are generally downregulated (16, 17). TGF-β has been frequently reviewed in tissue repair and fibrosis; it is known to be critical in wound healing, too (18). Similarly, IL-10 is essential to avoid excessive damage by limiting the inflammatory process (19). Neutrophils start to recede after pro-inflammatory factors (e.g., DAMPs) disappear and anti-inflammatory cytokines such as IL-4 and IL-10 accumulate (12, 15). Some neutrophils undergo apoptosis, whereas others leave the inflammatory site and re-enter the circulatory bloodstream, passing through lung capillaries on their way to the bone marrow where apoptosis occurs. Signals released from the apoptotic neutrophils recruit monocytes from the bone marrow to the injured site. They also stimulate macrophage transformation into the pro-resolving M2 phenotype. The clearance of neutrophil apoptotic bodies by macrophages contributes to tissue resolution. The activation of M2 macrophages is critical during the initial steps of the wound-healing process (20). The key cytokines for M2 macrophage activation are IL-4 and IL-13, which are produced by various cell types like mast cells and Th2 cells. Rheumatoid arthritis (RA) animal study proposed that MA can effectively stimulate adaptive immune cytokines, such as IL-4 and IL-13, to repair RA damage (21). Further statistical analysis of the cytokines and immune cells suggested that monocytes/macrophages, ECs, and several lymphocytes were the main cells affected by MA.

##### 2.1.1.3. Macrophages

Macrophages have long been implicated in the anti-inflammatory responses to acupuncture. Macrophage polarization is a complicated process and its role in inflammation is context- and time-dependent (15, 22). The classical M1 macrophage phenotype is activated by the presence of lipopolysaccharide (LPS)- or interferon-gamma (IFN-γ)-induced Th1 cells. M1 macrophages amplify the acute inflammatory signal by secreting cytokines such as tumor necrosis factor-alpha (TNF-α), IL-1β, IL-6, IL-12, and IL-23, as well as chemokines, NO, and ROS. Polarization to the anti-inflammatory M2 phenotype depends on Th2-secreted cytokines, including IL-4, IL-10, and IL-13. Acupuncture at ST36 downregulated M1-related cytokines (TNF-α, IL-1β, IL-6, IL-18) and increased the M2 population in different RA animal models (17). In a dextran sulfate sodium-induced murine colitis model, acupuncture relieved colitis symptoms by suppressing pro-inflammatory NLRP3/IL-1β, and promoting both the antioxidant Nrf2/HO-1 and the M1 to M2 transition (23). Acupuncture at the acupoints GV14, BL12, and BL13 alleviated airway hyper-responsiveness and mucus secretion in a murine model of asthma by balancing the M1/M2 and regulatory T cell (Treg)/Th17 cell populations (24). Similar anti-inflammatory effects have been described in various disease models, including Alzheimer's disease, ischemia-reperfusion injury, and RA (25–27). Additionally, macrophages from different sources play distinct roles in tissue repair. Among them, tissue-resident macrophages exhibit better regenerative capacity than those differentiated from recruited monocytes. A future study relating to acupoint stimulation of resident M2 transition may contribute to our understanding of its regenerative effects on injured tissues.

#### 2.1.2. The local inflammatory response—Mechanical stress-induced

Acupoints are usually rich in collagen fibers, vessels, and nerves. Traditional acupuncture practice includes needle rotation, which causes connective tissue deformation and influences stretch-sensitive cells.

##### 2.1.2.1. Fibroblasts

Fibroblasts are sentinels to both stretching and injury. Fibroblasts are the main resident cell population in the dermis, whose primary role is to produce an extracellular matrix that is crucial for skin structure (28). Single-cell analysis revealed that fibroblasts at a single anatomical location display great diversity, with specialized functional compartments. Dermal fibroblasts are stimulated by acupuncture both mechanically and biochemically. Needle rotation causes soft tissue deformation and cytoskeletal remodeling. Spindle-shaped fibroblasts transition into myofibroblasts upon tissue injury, with a flatter cell shape and more lamellipodia spreading in multiple directions (29). Myofibroblasts are mostly studied in fibrosis, in which connective tissues replace cellular compartments (30). However, little is known about the anti-fibrotic mechanisms involved in controlled wound healing.

##### 2.1.2.2. Mast cells

Mast cells are mainly distributed in connective tissues that are exposed to the external environment (31). Mast cells in healthy adult skin are mostly located in the dermis and near blood vessels, nerves, and hair follicles, especially in the distal areas such as the arms and legs as compared to the proximal areas. Some mast cells line the luminal layers of vessels, aligning with endothelial cells. The classical activation pathway of mast cells involves binding of allergens to specific immunoglobulin-E receptor. This leads to the degranulation of histamines, proteases, and cytokines, and *de novo* synthesis of inflammatory lipids (prostaglandins and leukotrienes). A broader spectrum of chemokines, cytokines, and growth factors is then upregulated in the mast-cell mediated allergic reactions. Some mast cell signaling molecules travel longer distances *via* exosomes (32). Ion channels like the transient receptor potential (TRP) family are being investigated as mediators of allergic reactions (33). Mast cells are known to express TRPV channels. As some of the family members are activated by mechanical stress, mast cells may play a considerable role in MA-induced microenvironment changes (34). Blockage of TRPV4 channels hindered the analgesic effect of acupuncture at acupoint ST36 in a rat model of RA (35). In addition to their role in mechano-sensing, mast cells are involved in tissue repair and skin remodeling by interacting with fibroblasts. Human mast cells secrete several classes of fibroblast growth factors, which can stimulate the differentiation and proliferation of dermal fibroblasts (36). As mentioned previously, proper fibroblast activation is essential for ideal wound healing. A detailed study of the relationship between acupuncture and matrix protein deposition may contribute to the prevention of fibrosis.

### 2.2. Crosstalk between the nervous system and the immune system

Recent studies have made great progress in establishing neural mechanisms underlying acupuncture-mediated immune modulation. Acupuncture is able to activate the vagal afferent nerves. Electric stimulation of the vagus nerve dampens endotoxin-induced systemic inflammation, and vagotomy attenuates acupuncture effects (37–39). Analgesia is another topic of interest in acupuncture. The relationship between nociception and immunity is beginning to be elucidated.

#### 2.2.1. Vagus nerve-dependent pathway

Homeostasis of systemic immunity is modulated by neural reflex circuits (40). Communication between the central nervous system (CNS) and peripheral tissues is mainly carried by the vagus nerve. The vagus nerve innervates the organs in the thorax and abdomen and is known to be crucial for internal environment regulation *via* acupuncture (41). Afferent signals from the vagus nerve are processed by the brainstem, and efferent signals travel down to target organs, such as the adrenal glands and spleen. To date, three pathways are known to be associated with acupuncture: the cholinergic anti-inflammatory pathway (CAIP), vagus-adrenal pathway, and splenic sympathetic pathway.

##### 2.2.1.1. The cholinergic anti-inflammatory system

Both the preganglionic and post-ganglionic nerves are cholinergic in the parasympathetic system. Acetylcholine (Ach) is the primary neurotransmitter released at synapses in the ganglion. Cholinergic receptors include nicotinic receptors (nAChRs) and muscarinic receptors. The anti-inflammatory effect of the cholinergic system mainly relies on the α7nAChR. The upregulation of α7nAChR is negatively correlated with systemic pro-inflammatory cytokine levels in several inflammatory diseases (37). In endotoxemia, electrical stimulation of the vagus nerve downregulates TNF-producing macrophages in the spleen in an α7nAChR-dependent manner. Immune regulation by the cholinergic system is organ-specific. In contrast to the spleen, EA ameliorates chronic obstructive pulmonary disease and asthma symptoms by downregulating the cholinergic system (17). Further studies are needed to unravel how different acupoint stimulations affect the CAIP in different organs.

##### 2.2.1.2. The vagus–adrenal axis

The HPA axis is known for its immunosuppressive function of releasing catecholamines into the bloodstream (42). The paraventricular nucleus of the hypothalamus integrates inputs from different neural circuits, including the sympathetic and parasympathetic circuits. Different neural network activations lead to the release of various catecholamines. EA at ST36 activates the sciatic nerve, which sends signals to the vagus nerve, resulting in dopamine release from the adrenal medulla (11). This mechanism depends solely on dopamine type 1 (D1) receptors. Neither β2 adrenoceptors (β2ARs) nor α7nAChR knockout diminished dopamine release and the anti-inflammatory effect of EA. The intensity of the current seems to be critical, as only low-intensity (0.5 mA) EA at ST36 activated the vagus**–**adrenal axis (43). Stronger current intensities primarily activated the splenic sympathetic axis.

##### 2.2.1.3. The splenic sympathetic nerve

Sympathetic regulation of systemic immunity is closely related to norepinephrine (NE) and adrenergic receptors (ARs) (44). In LPS-induced sepsis models, electric stimulation of the splenic nerve induced NE secretion from the spleen. NE suppressed TNF production by regulating β2 AR-expressing cells (45). The sympathetic nerves can also mediate pro-inflammatory effects if α2 ARs are dominant (46). Notably, research by Ma et al. has underlined the importance of prior health conditions in EA-mediated therapeutic effects. Different nerve fibers are innervated by different acupoints. Since current neuronal studies have focused on very few acupoints, more research is required to whether these pathways can be activated by other acupoints with similar therapeutic effects.

#### 2.2.2. Pain in immune regulation

Pain sensations are generated by a group of sensory neurons that express nociceptors. It is generally accepted that inflammation causes pain through sensitizing nociceptor neurons (47). Mast cells play a major role in sensitizing nociceptor sensory neurons during both acute and chronic inflammation (48). Mast cell activation can sensitize neighboring sensory neurons, which is sometimes called “acupoint sensitization” (49). Neurons become hyper-reactive upon local accumulation of allergic substances and neuropeptides such as substance P, histamine, serotonin, and tryptase. Meanwhile, the nerve growth factors produced by mast cells can increase local nociceptor density, which increases the tissue's pain sensitivity (50, 51). Other immune cells, such as macrophages, can also modify ion channels on neurons to increase firing rates.

Pain sensations caused by nociceptors influence neighboring vasculature and immune cells. Vasodilation directly induced by electrical stimulation is called neurogenic inflammation (52). This process is dependent on substance P and calcitonin gene-related peptide (CGRP), whose release causes vasodilation (53). Many immune cells express receptors for neuropeptides and neurotransmitters released from nociceptor neuron terminals (47). Consistently, denervation leads to better outcomes in conditions caused by dysregulated inflammation such as chronic obstructive pulmonary disease and arthritis (54, 55). In contrast, nociceptor neurons can also dampen innate immunity under certain conditions, such as endotoxemia (56). Pretreatment with CGRP reduced mortality, in association with reduced TNF-α and upregulated IL-10 production by macrophages.

However, the neuroimmune modulatory function of acupuncture remains largely unknown. This may be associated with its analgesic effect. Several hypotheses have been made regarding the mechanism involved in acupuncture analgesia; thus far, the microglial cells and astrocytes in the central nervous system and the endogenous opioid system have been suggested to interfere with pain signals (16). The gate control theory has also been introduced in acupuncture analgesia. This theory claims that stimulation of non-nociceptive Aβ sensory fibers by needle insertion activates the inhibitory dorsal horn interneuron, which cut off the incoming pain signal from the Aδ and C nerve fibers (57, 58). Linking the molecular basis of acupuncture analgesia to that of acupuncture-mediated immunomodulation will provide a new perspective in this field.

## 3. Discussion

We unfolded the local immune response mediated by acupuncture by using the term “sterile inflammation.” The participants of sterile inflammation are rather variable: intracellular DAMPs and extracellular DAMPs are recognized by different sets of receptors (12). Macrophages originate from different pools seem to play differential roles during inflammation. The heart damage repaired by the embryonic-derived macrophages was scar-free in neonatal mice. The study shows that fibrosis occurs when the monocyte-derived macrophages were involved in the tissue resolution, while the inhibition of this macrophage pool resulted in better outcomes (15, 59). The profiling of the key signaling molecules and cell types, and the origins of macrophages after the acupuncture treatment will better elaborate its local immunomodulatory effect.

Stimulation of acupoints at extremities can modulate the systemic inflammatory status. The anti-inflammatory effect of ST36 stimulation is well-studied in sepsis and colitis models. The vagus nerve has been put at the center of the neural study of acupuncture. Recent studies published by Dr. Ma (43, 44) successfully figured out the different reflexes activated by electroacupuncture of different frequencies at different acupoints. Further mapping of neural networks activated upon electric stimulation of different acupoints may provide information about how the nervous system modulates one's immune system upon different afferent signals.

The crosstalk between the two systems mentioned in this review has been focused on pain. Mast cells can be activated upon both chemical and mechanical stimulation, releasing allergic substances and neuropeptides that can sensitize the surrounding nociceptors. Electric stimulation of the pain-sensing neurons can either upregulate or downregulate local inflammation, which seems to depend on the health condition. On the other hand, acupuncture treatment has been reported to change the host gut microbiome in certain disease models. EA was protective against the deleterious metabolic changes caused by high-fat diet-induced obesity *via* modulating the gut microbiota (60). MA mitigated the gut microbiota changes in the APP/PS1 Alzheimer's disease mouse group, and the cognitive function and the intestinal barriers were also improved in the MA treatment group (61). The gut microbiota is another potential platform to explain the crosstalk between the immune system and the neural system.

**Figure 1:**
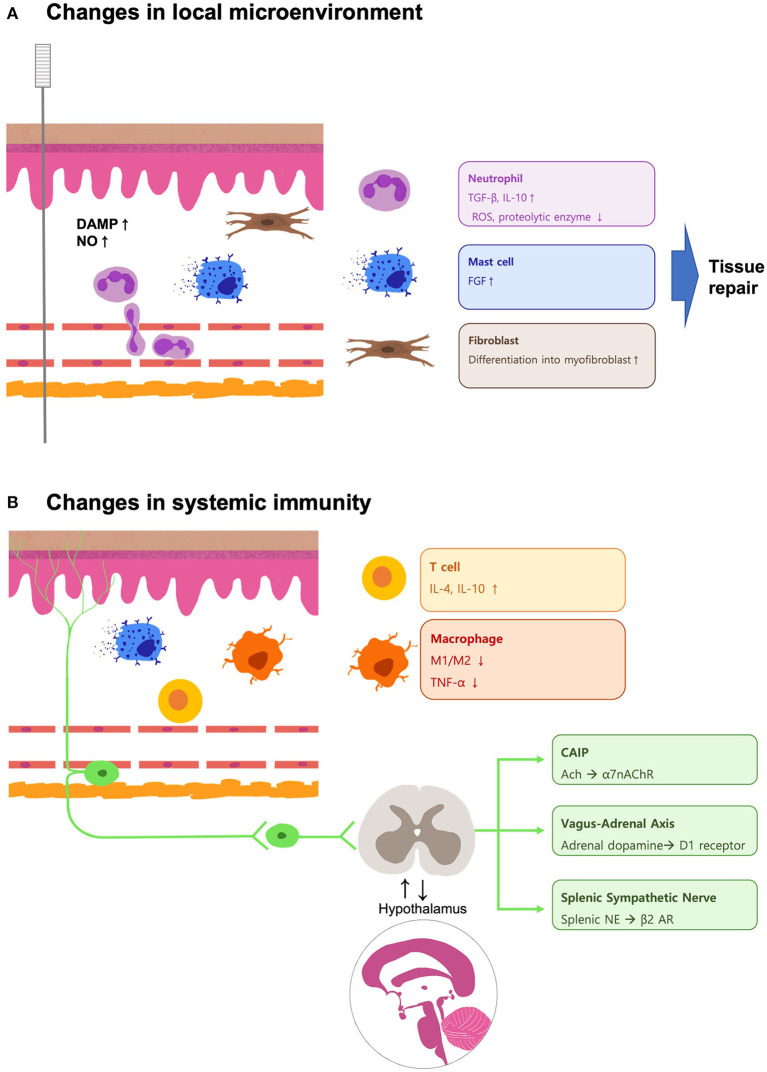
Schematic illustration of the mechanisms involved in the acupuncture-mediated changes in the local microenvironment and systemic immunity during inflammation. **(A)** The local DAMPs and NO accumulate upon the needle insertion, and the leukocytes like neutrophils are recruited. The neutrophils secrete tissue-resolving cytokines like TGF-*β* and IL-10, while ROS and proteolytic enzymes are downregulated during the sterile inflammation. The mechanical stress caused by the needle rotation triggers the activation of mast cells and fibroblasts, which participate in the wound-healing process. **(B)** The concentration of DAMPs decreases, and the neutrophils undergo apoptosis. Acupuncture treatment promotes tissue resolution by balancing the local Treg/Th17 ratio. Increased concentrations of IL-4, IL-10, and IL-13 drive the shifting of macrophages to M2. The signal transduced by the neurons may provide a postponed systemic anti-inflammatory effect by upregulation of the CAIP, vagus–adrenal axis, and splenic sympathetic nerve. These pathways are also involved in acupuncture analgesia. These findings implicate the interaction between the nervous system and the immune system to achieve the therapeutic effects mediated by acupuncture.

In this review, we highlighted the changes in the local microenvironment mediated by acupuncture stimulation and its systemic effects during inflammation (Figure 1). Biological and mechanical changes induced by acupuncture result in various immune responses in ECs, neutrophils, macrophages, and mast cells. These responses induce systemic immunity *via* neuroimmune mechanisms, such as the cholinergic, vagal-adrenal, and splenic sympathetic nervous pathways. Pain modulation and its neuroimmune mechanism are also promising for unraveling the inflammation-related mechanisms of acupuncture. Further research is needed to explore mechanisms linked to the diverse inflammation-related effects of acupuncture.

## Author contributions

W-LY wrote the manuscript. J-YP and H-JP revised the manuscript. S-NK supervised the study. All authors contributed to the article and approved the submitted version.

## References

[1] World Health Organization Regional Office for the Western Pacific. WHO Standard Acupuncture Point Locations in the Western Pacific Region.: WHO Regional Office for the Western Pacific. (2008). Available online at: https://apps.who.int/iris/handle/10665/353407 (accessd July 7, 2022).

[2] Chavez LM Huang SS MacDonald I Lin JG Lee YC Chen YH. Mechanisms of acupuncture therapy in ischemic stroke rehabilitation: a literature review of basic studies. Int J Mol Sci. (2017) 18:2270. 10.3390/ijms18112270 29143805 PMC5713240

[3] Feng Y Fang Y Wang Y Hao Y. Acupoint therapy on diabetes mellitus and its common chronic complications: a review of its mechanisms. Biomed Res Int. (2018) 2018:3128378. 10.1155/2018/3128378 30426006 PMC6217896

[4] Wang M Gao YH Xu J Chi Y Wei XB Lewith G. Zusanli (ST36) acupoint injection for preventing post-operative ileus: a systematic review and meta-analysis of randomized clinical trials. Complement Ther Med. (2015) 23:469–83. 10.1016/j.ctim.2015.03.013 26051583 PMC4909358

[5] Li L Zang H Jiang Y Zhang Y Mu S Cao J. Acupuncture at back-shu and front-mu acupoints prevents gastric ulcer by regulating the TLR4/MyD88/NF-kappaB signaling pathway. Evid Based Complem Alternat Med. (2021) 2021:8214052. 10.1155/2021/8214052 33628315 PMC7886517

[6] Wang L Yang JW Lin LT Huang J Wang XR Su XT. Acupuncture attenuates inflammation in microglia of vascular dementia rats by inhibiting mir-93-mediated TLR4/MyD88/NF-kappaB signaling pathway. Oxid Med Cell Longev. (2020) 2020:8253904. 10.1155/2020/8253904 32850002 PMC7441436

[7] Zhang CN Huang XK Luo Y Jiang J Wan L Wang L. The effects of electro-acupuncture on the signaling pathway of TLR/MYD88 in ankle joint synovial tissue of acute gouty arthritis rats. Sichuan Da Xue Xue Bao Yi Xue Ban. (2014) 45:924–7. 25571716

[8] da Silva MD Bobinski F Sato KL Kolker SJ Sluka KA Santos AR. IL-10 cytokine released from M2 macrophages is crucial for analgesic and anti-inflammatory effects of acupuncture in a model of inflammatory muscle pain. Mol Neurobiol. (2015) 51:19–31. 10.1007/s12035-014-8790-x 24961568 PMC4276737

[9] Nurwati I Muthmainah M Huda KN. Acupuncture for asthma: its potential significance in clinical practice. Med Acupunct. (2020) 32:272–9. 10.1089/acu.2020.1443 33101571 PMC7583338

[10] Dou B Li Y Ma J Xu Z Fan W Tian L. Role of Neuroimmune crosstalk in mediating the anti-inflammatory and analgesic effects of acupuncture on inflammatory pain. Front Neurosci. (2021) 15:695670. 10.3389/fnins.2021.695670 34408622 PMC8366064

[11] Torres-Rosas R Yehia G Pena G Mishra P. del Rocio Thompson-Bonilla M Moreno-Eutimio MA. Dopamine mediates vagal modulation of the immune system by electroacupuncture. Nat Med. (2014) 20:291–5. 10.1038/nm.3479 24562381 PMC3949155

[12] Zindel J Kubes P. DAMPs, PAMPs, and LAMPs in immunity and sterile inflammation. Annu Rev Pathol. (2020) 15:493–518. 10.1146/annurev-pathmechdis-012419-032847 31675482

[13] Félétou M. The Endothelium: Part 1: Multiple Functions of the Endothelial Cells—Focus on Endothelium-Derived Vasoactive Mediators. San Rafael, CA: Morgan & Claypool Life Sciences (2011). 10.4199/C00031ED1V01Y201105ISP019 21850763

[14] Ma SX. Nitric oxide signaling molecules in acupoints: toward mechanisms of acupuncture. Chin J Integr Med. (2017) 23:812–5. 10.1007/s11655-017-2789-x 29080196 PMC5761672

[15] Eming SA Wynn TA Martin P. Inflammation and metabolism in tissue repair and regeneration. Science. (2017) 356:1026–30. 10.1126/science.aam7928 28596335

[16] Chen T Zhang WW Chu YX Wang YQ. Acupuncture for pain management: molecular mechanisms of action. Am J Chin Med. (2020) 48:793–811. 10.1142/S0192415X20500408 32420752

[17] Li N Guo Y Gong Y Zhang Y Fan W Yao K. The anti-inflammatory actions and mechanisms of acupuncture from acupoint to target organs *via* neuro-immune regulation. J Inflamm Res. (2021) 14:7191–224. 10.2147/JIR.S341581 34992414 PMC8710088

[18] Morikawa M Derynck R Miyazono K. TGF-beta and the TGF-beta family: context-dependent roles in cell and tissue physiology. Cold Spring Harb Perspect Biol. (2016) 8:a021873. 10.1101/cshperspect.a021873 27141051 PMC4852809

[19] Ouyang W O'Garra A. IL-10 family cytokines IL-10 and IL-22: from basic science to clinical translation. Immunity. (2019) 50:871–91. 10.1016/j.immuni.2019.03.020 30995504

[20] Van Dyken SJ Locksley RM. Interleukin-4- and interleukin-13-mediated alternatively activated macrophages: roles in homeostasis and disease. Annu Rev Immunol. (2013) 31:317–43. 10.1146/annurev-immunol-032712-095906 23298208 PMC3606684

[21] Zhang K Zhao X Ding S Liu Y Xu Y Yan Y. Applying complex network and cell-cell communication network diagram methods to explore the key cytokines and immune cells in local acupoint involved in acupuncture treating inflammatory pain. Evid Based Complement Alternat Med. (2020) 2020:2585960. 10.1155/2020/2585960 32802117 PMC7411476

[22] Wynn TA Vannella KM. Macrophages in tissue repair, regeneration, and fibrosis. Immunity. (2016) 44:450–62. 10.1016/j.immuni.2016.02.015 26982353 PMC4794754

[23] Song S An J Li Y Liu S. Electroacupuncture at ST-36 ameliorates DSS-induced acute colitis *via* regulating macrophage polarization induced by suppressing NLRP3/IL-1beta and promoting Nrf2/HO-1. Mol Immunol. (2019) 106:143–52. 10.1016/j.molimm.2018.12.023 30610999

[24] Dong M Wang WQ Chen J Li MH Xu F Cui J. Acupuncture regulates the balance of CD4(+) T cell subtypes in experimental asthma mice. Chin J Integr Med. (2019) 25:617–24. 10.1007/s11655-018-3055-6 30519873

[25] Liu R Xu NG Yi W Ji C. Electroacupuncture attenuates inflammation after ischemic stroke by inhibiting NF-kappaB-mediated activation of microglia. Evid Based Complement Alternat Med. (2020) 2020:8163052. 10.1155/2020/8163052 32922507 PMC7453260

[26] Wang X Li Z Li C Wang Y Yu S Ren L. Electroacupuncture with Bushen Jiannao improves cognitive deficits in senescence-accelerated mouse prone 8 mice by inhibiting neuroinflammation. J Tradit Chin Med. (2020) 40:812–9. 10.19852/j.cnki.jtcm.2020.05.011 33000582

[27] Yu ML Wei RD Zhang T Wang JM Cheng Y Qin FF. Electroacupuncture relieves pain and attenuates inflammation progression through inducing IL-10 production in CFA-induced mice. Inflammation. (2020) 43:1233–45. 10.1007/s10753-020-01203-2 32198725

[28] Lynch MD Watt FM. Fibroblast heterogeneity: implications for human disease. J Clin Invest. (2018) 128:26–35. 10.1172/JCI93555 29293096 PMC5749540

[29] Langevin HM Bouffard NA Badger GJ Churchill DL Howe AK. Subcutaneous tissue fibroblast cytoskeletal remodeling induced by acupuncture: evidence for a mechanotransduction-based mechanism. J Cell Physiol. (2006) 207:767–74. 10.1002/jcp.20623 16511830

[30] Gerarduzzi C Di Battista JA. Myofibroblast repair mechanisms post-inflammatory response: a fibrotic perspective. Inflamm Res. (2017) 66:451–65. 10.1007/s00011-016-1019-x 28040859

[31] Voss M Kotrba J Gaffal E Katsoulis-Dimitriou K Dudeck A. Mast cells in the skin: defenders of integrity or offenders in inflammation? Int J Mol Sci. (2021) 22:4589. 10.3390/ijms22094589 33925601 PMC8123885

[32] Valadi H Ekstrom K Bossios A Sjostrand M Lee JJ Lotvall JO. Exosome-mediated transfer of mRNAs and microRNAs is a novel mechanism of genetic exchange between cells. Nat Cell Biol. (2007) 9:654–9. 10.1038/ncb1596 17486113

[33] Nam JH Kim WK. The role of TRP channels in allergic inflammation and its clinical relevance. Curr Med Chem. (2020) 27:1446–68. 10.2174/0929867326666181126113015 30474526

[34] Wang LN Wang XZ Li YJ Li BR Huang M Wang XY. Activation of subcutaneous mast cells in acupuncture points triggers analgesia. Cells. (2022) 11:809. 10.3390/cells11050809 35269431 PMC8909735

[35] Zheng Y Zuo W Shen D Cui K Huang M Zhang D. Mechanosensitive TRPV4 channel-induced extracellular ATP accumulation at the acupoint mediates acupuncture analgesia of ankle arthritis in rats. Life. (2021) 11:513. 10.3390/life11060513 34073103 PMC8228741

[36] Artuc M Steckelings UM Henz BM. Mast cell-fibroblast interactions: human mast cells as source and inducers of fibroblast and epithelial growth factors. J Invest Dermatol. (2002) 118:391–5. 10.1046/j.0022-202x.2001.01705.x 11874475

[37] Rosas-Ballina M Ochani M Parrish WR Ochani K Harris YT Huston JM. Splenic nerve is required for cholinergic antiinflammatory pathway control of TNF in endotoxemia. Proc Natl Acad Sci USA. (2008) 105:11008–13. 10.1073/pnas.0803237105 18669662 PMC2504833

[38] Huston JM Ochani M Rosas-Ballina M Liao H Ochani K Pavlov VA. Splenectomy inactivates the cholinergic antiinflammatory pathway during lethal endotoxemia and polymicrobial sepsis. J Exp Med. (2006) 203:1623–8. 10.1084/jem.20052362 16785311 PMC2118357

[39] Borovikova LV Ivanova S Zhang M Yang H Botchkina GI Watkins LR. Vagus nerve stimulation attenuates the systemic inflammatory response to endotoxin. Nature. (2000) 405:458–62. 10.1038/35013070 10839541

[40] Andersson U Tracey KJ. Reflex principles of immunological homeostasis. Annu Rev Immunol. (2012) 30:313–35. 10.1146/annurev-immunol-020711-075015 22224768 PMC4533843

[41] Lim HD Kim KJ Jo BG Park JY Namgung U. Acupuncture stimulation attenuates TNF-alpha production *via* vagal modulation in the concanavalin a model of hepatitis. Acupunct Med. (2020) 38:417–25. 10.1177/0964528420907338 32233774

[42] Silverman MN Sternberg EM. Glucocorticoid regulation of inflammation and its functional correlates: from HPA axis to glucocorticoid receptor dysfunction. Ann N Y Acad Sci. (2012) 1261:55–63. 10.1111/j.1749-6632.2012.06633.x 22823394 PMC3572859

[43] Liu S Wang Z Su Y Qi L Yang W Fu M. A neuroanatomical basis for electroacupuncture to drive the vagal-adrenal axis. Nature. (2021) 598:641–5. 10.1038/s41586-021-04001-4 34646018 PMC9178665

[44] Ma Q. Somato-autonomic reflexes of acupuncture. Med Acupunct. (2020) 32:362–6. 10.1089/acu.2020.1488 33362888 PMC7755849

[45] Liu S Wang ZF Su YS Ray RS Jing XH Wang YQ. Somatotopic organization and intensity dependence in driving distinct NPY-expressing sympathetic pathways by electroacupuncture. Neuron. (2020) 108:436–50e7. 10.1016/j.neuron.2020.07.015 32791039 PMC7666081

[46] Huang JL Zhang YL Wang CC Zhou JR Ma Q Wang X. Enhanced phosphorylation of MAPKs by NE promotes TNF-alpha production by macrophage through alpha adrenergic receptor. Inflammation. (2012) 35:527–34. 10.1007/s10753-011-9342-4 21590324

[47] Pinho-Ribeiro FA Verri WA Chiu IM. Nociceptor sensory neuron-immune interactions in pain and inflammation. Trends Immunol. (2017) 38:5–19. 10.1016/j.it.2016.10.001 27793571 PMC5205568

[48] Oliveira SM Drewes CC Silva CR Trevisan G Boschen SL Moreira CG. Involvement of mast cells in a mouse model of post-operative pain. Eur J Pharmacol. (2011) 672:88–95. 10.1016/j.ejphar.2011.10.001 22004612

[49] Tan H Tumilty S Chapple C Liu L McDonough S Yin H. Understanding acupoint sensitization: a narrative review on phenomena, potential mechanism, and clinical application. Evid Based Complement Alternat Med. (2019) 2019:6064358. 10.1155/2019/6064358 31485246 PMC6710800

[50] Leon A Buriani A Dal Toso R Fabris M Romanello S Aloe L. Mast cells synthesize, store, and release nerve growth factor. Proc Natl Acad Sci U S A. (1994) 91:3739–43. 10.1073/pnas.91.9.3739 8170980 PMC43657

[51] Denk F Bennett DL McMahon SB. Nerve growth factor and pain mechanisms. Annu Rev Neurosci. (2017) 40:307–25. 10.1146/annurev-neuro-072116-031121 28441116

[52] Jancso N Jancso-Gabor A Szolcsanyi J. Direct evidence for neurogenic inflammation and its prevention by denervation and by pretreatment with capsaicin. Br J Pharmacol Chemother. (1967) 31:138–51. 10.1111/j.1476-5381.1967.tb01984.x 6055248 PMC1557289

[53] Schlereth T Schukraft J Kramer-Best HH Geber C Ackermann T Birklein F. Interaction of calcitonin gene related peptide (CGRP) and substance P (SP) in human skin. Neuropeptides. (2016) 59:57–62. 10.1016/j.npep.2016.06.001 27344069

[54] Aaron SD Mahler DA. Calming nervous airways: targeted lung denervation for chronic obstructive pulmonary disease. Am J Respir Crit Care Med. (2019) 200:1455–6. 10.1164/rccm.201908-1586ED 31469579 PMC6909830

[55] Peltz TS Yapp LZ Elherik FK Breusch SJ. Patient satisfaction and outcomes of partial wrist denervation in inflammatory arthritis. Clin Rheumatol. (2019) 38:2995–3003. 10.1007/s10067-019-04645-8 31290023

[56] Gomes RN Castro-Faria-Neto HC Bozza PT Soares MB Shoemaker CB David JR. Calcitonin gene-related peptide inhibits local acute inflammation and protects mice against lethal endotoxemia. Shock. (2005) 24:590–4. 10.1097/01.shk.0000183395.29014.7c 16317392

[57] Melzack R Wall PD. Pain mechanisms: a new theory. Science. (1965) 150:971–9. 10.1126/science.150.3699.971 5320816

[58] Moayedi M Davis KD. Theories of pain: from specificity to gate control. J Neurophysiol. (2013) 109:5–12. 10.1152/jn.00457.2012 23034364

[59] Lavine KJ Epelman S Uchida K Weber KJ Nichols CG Schilling JD. Distinct macrophage lineages contribute to disparate patterns of cardiac recovery and remodeling in the neonatal and adult heart. Proc Natl Acad Sci USA. (2014) 111:16029–34. 10.1073/pnas.1406508111 25349429 PMC4234568

[60] Wang H Wang Q Liang C Su M Wang X Li H. Acupuncture regulating gut microbiota in abdominal obese rats induced by high-fat diet. Evid Based Complement Alternat Med. (2019) 2019:4958294. 10.1155/2019/4958294 31275411 PMC6582896

[61] Hao X Ding N Zhang Y Yang Y Zhao Y Zhao J. Benign regulation of the gut microbiota: the possible mechanism through which the beneficial effects of manual acupuncture on cognitive ability and intestinal mucosal barrier function occur in APP/PS1 mice. Front Neurosci. (2022) 16:960026. 10.3389/fnins.2022.960026 35992924 PMC9382294

